# Traditional Uses, Pharmacological Effects, and Molecular Mechanisms of Licorice in Potential Therapy of COVID-19

**DOI:** 10.3389/fphar.2021.719758

**Published:** 2021-11-26

**Authors:** Qian-hui Zhang, Hao-zhou Huang, Min Qiu, Zhen-feng Wu, Zhan-chang Xin, Xin-fu Cai, Qiang Shang, Jun-zhi Lin, Ding-kun Zhang, Li Han

**Affiliations:** ^1^ State Key Laboratory of Southwestern Chinese Medicine Resources, Pharmacy School, Chengdu University of Traditional Chinese Medicine, Chengdu, China; ^2^ Jiangxi University of Traditional Chinese Medicine, Nanchang, China; ^3^ Gansu Qilian Mountain Pharmaceutical Limited Liability Company, Jiuquan, China; ^4^ Sichuan Guangda Pharmaceutical Co. Ltd, Pengzhou, China; ^5^ National Engineering Research Center for Modernization of Traditional Chinese Medicine, Pengzhou, China; ^6^ TCM Regulating Metabolic Diseases Key Laboratory of Sichuan Province, Hospital of Chengdu University of Traditional Chinese Medicine, Chengdu, China

**Keywords:** COVID-19, licorice, traditional uses, pharmacological effects, mechanism of action

## Abstract

The current Coronavirus disease 2019 (COVID-19) pandemic has become a global challenge, and although vaccines have been developed, it is expected that mild to moderate patients will control their symptoms, especially in developing countries. Licorice, not only a food additive, but also a common traditional Chinese herbal medicine, which has several pharmacological effects, such as anti-inflammation, detoxification, antibacterial, antitussive, and immunomodulatory effects, especially in respiratory diseases. Since the outbreak of COVID-19, glycyrrhizin, glycyrrhizin diamine and glycyrrhizin extract have been widely studied and used in COVID-19 clinical trials. Therefore, it is a very interesting topic to explore the material basis, pharmacological characteristics and molecular mechanism of licorice in adjuvant treatment of COVID-19. In this paper, the material basis of licorice for the prevention and treatment of COVID-19 is deeply analyzed, and there are significant differences among different components in different pharmacological mechanisms. Glycyrrhizin and glycyrrhetinic acid inhibit the synthesis of inflammatory factors and inflammatory mediators by blocking the binding of ACE 2 to virus spike protein, and exert antiviral and antibacterial effects. Immune cells are stimulated by multiple targets and pathways to interfere with the pathogenesis of COVID-19. Liquiritin can prevent and cure COVID-19 by simulating type I interferon. It is suggested that licorice can exert its therapeutic advantage through multi-components and multi-targets. To sum up, licorice has the potential to adjuvant prevent and treat COVID-19. It not only plays a significant role in anti-inflammation and anti-ACE-2, but also significantly improves the clinical symptoms of fever, dry cough and shortness of breath, suggesting that licorice is expected to be a candidate drug for adjuvant treatment of patients with early / mild COVID-19.

## Introduction

Since the outbreak of COVID-19 (December 12, 2019), there has been more than 240.34 million confirmed cases, and the epidemic situation is still grim all over the world. In view of the fact that it will take time for COVID-19 vaccine to be widely vaccinated all over the world, especially the great threat posed by virus mutation to people in developing countries, there is an urgent need to find safe complementary and alternative treatments to make up for the gap in vaccination temporarily. In addressing the COVID-19 epidemic around the world, the integration of traditional Chinese and Western medicine has become a significant feature of the “China plan.” Traditional Chinese medicine has been used in the prevention and treatment of epidemic diseases for thousands of years, from smallpox and ancient plagues to avian influenza, middle east respiratory syndrome (MERS), severe acute respiratory syndrome (SARS), and so on (Lin et al., 2017). According to Stanford University scholars and researchers at the University of Hong Kong, severe acute respiratory syndrome coronavirus 2 (SARS-CoV2) enters the cells through the same path as the SARS coronavirus, that is to say, by the angiotensin-converting enzyme 2 (ACE2) cell receptor, which proves that glycyrrhizin can bind to part of the binding site of ACE2, indicating that glycyrrhizin has potential application for inhibiting SARS-CoV2 (Zhou P. et al., 2020). Meanwhile, the other study has mentioned that glycyrrhizin could significantly prevent the binding of novel coronavirus spikes protein to the human target protein ACE2, confirming that glycyrrhizin is an effective bioactive components against COVID-19 (Yu et al., 2020). Additionally, according to a team at Peking University, liquiritin can inhibit COVID-19 by simulating type I interferon (ZhuJie et al., 2020). Therefore, it certainly has a reference point for exploring the mechanism and bioactive components of licorice in the treatment of COVID-19.

Licorice, the dried root and rhizome of the plant *Glycyrrhiza uralensis* Fisch*.* ex DC., *Glycorrhiza inflata* Bat*., Glycyrrhiza glabra* L*.* *.* It has various effects, including clearing heat and removing the toxin, relieving pain and cough, dispelling phlegm, and reconciling numerous medicines (Wang et al., 2019). Licorice has a long history of medicinal use in both Eastern and Western civilizations. In the West, Romans, Greeks, the scriptures of Ayurveda, and the ancient Egyptians mentioned the beneficial effects of licorice in traditional treatment of colds, coughs, and chills. For example, In IV-III century B.C., the Greeks first used licorice as a medicine in Europe to treat asthma, lung disease, and cough. In IV-V century A.D., licorice was used to relieve fever, influenza syndrome, nourish blood, and restore blood circulation (Wang J. et al., 2013). The Romans recommended licorice to treat lung diseases. Since the VIII-IX century A.D., licorice was used to relieve cough to treat various lung diseases (Fiore et al., 2005). Since the Middle Ages, Germans have used licorice to relieve diseases such as arterial disease, palpitations, and angina pectoris. In the East, it has a history of more than 2,000 years of medicinal use. It has been widely used to treat various diseases, such as respiratory diseases, fever, hypertension, gastric ulcers, paralysis, rheumatism, sexual weakness, and hemorrhagic diseases. According to the theory of traditional Chinese medicine, licorice has a unique conditioning effect on respiratory diseases, viral cough, viral hepatitis, and other diseases. Although licorice has many pharmacological effects, and often be used as a unique “guiding medicine” in more than half of the traditional and modern prescriptions and prescriptions. The regulatory effects of licorice on other herbs include significant detoxification, treatment of drug and food poisoning, or inhibition of adverse reactions, and this “guiding” effect has been tested in many formulations. In India, licorice is widely used to treat influenza, eye disease, gallstones, liver disease and arthritis (Zhang et al., 2020). In Japan, licorice is widely used to detoxify, relieve cough, and relieve pain. In the 1940s, Japanese pharmaceutical company Minuofa (Minophagen) Pharmaceutical Co., Ltd. successfully extracted glycyrrhizin from licorice and formed a compound preparation Stronger Neo-Minophagen C with glycine and cysteine, which has been used as anti-allergy and anti-hepatitis drugs in the clinic. Additionally, because licorice, as a monoamine oxidase inhibitor, has anticholinergic, antitussive, hypolipidemic, antifungal, antioxidant, and anticancer effects, it has traditionally been used as anti-inflammatory, anti-ulcer, antibiotic, anti-arthritic, antiviral, laxative, memory stimulant (Zadeh et al., 2013). Since the 18th century, licorice has been used in various food, industrial, pharmaceutical, and cosmetic applications, with sound therapeutic effects and high safety. Meanwhile, licorice is added to many foods as an important spice. In modern food production, licorice is widely used in beverages, beer, meat and other foods as sweeteners, antioxidants, antimicrobials, foaming agents and flavor enhancers. It is a recognized food additive in the Europe, the United States and China (Montoro et al., 2011; Di Lorenzo et al., 2015; Li et al., 2016; Xu et al., 2016; Alam et al., 2017; Indhu and Shajahan, 2018). Modern pharmacological studies have shown that licorice has numerous effects, such as anti-inflammatory, antiviral, antibacterial, and immunomodulatory, as well as certain hypoglycemic, anti-obesity, and detoxification effects (Yang et al., 2015; Luo et al., 2020). Licorice is used in Russian traditional and officinal medicine as an expectorant and emollient, which is related to anti-inflammatory properties. However, all medicines of licorice are available in Russia in Pharmacies as OTC products in European Union (Shikov Alexander et al., 2021). Among them, the present study showed that licorice could cure infection related ailments with the establishment of glabridin as a potent lead molecule for activity (Fatima et al., 2009).

**FIGURE 1:**
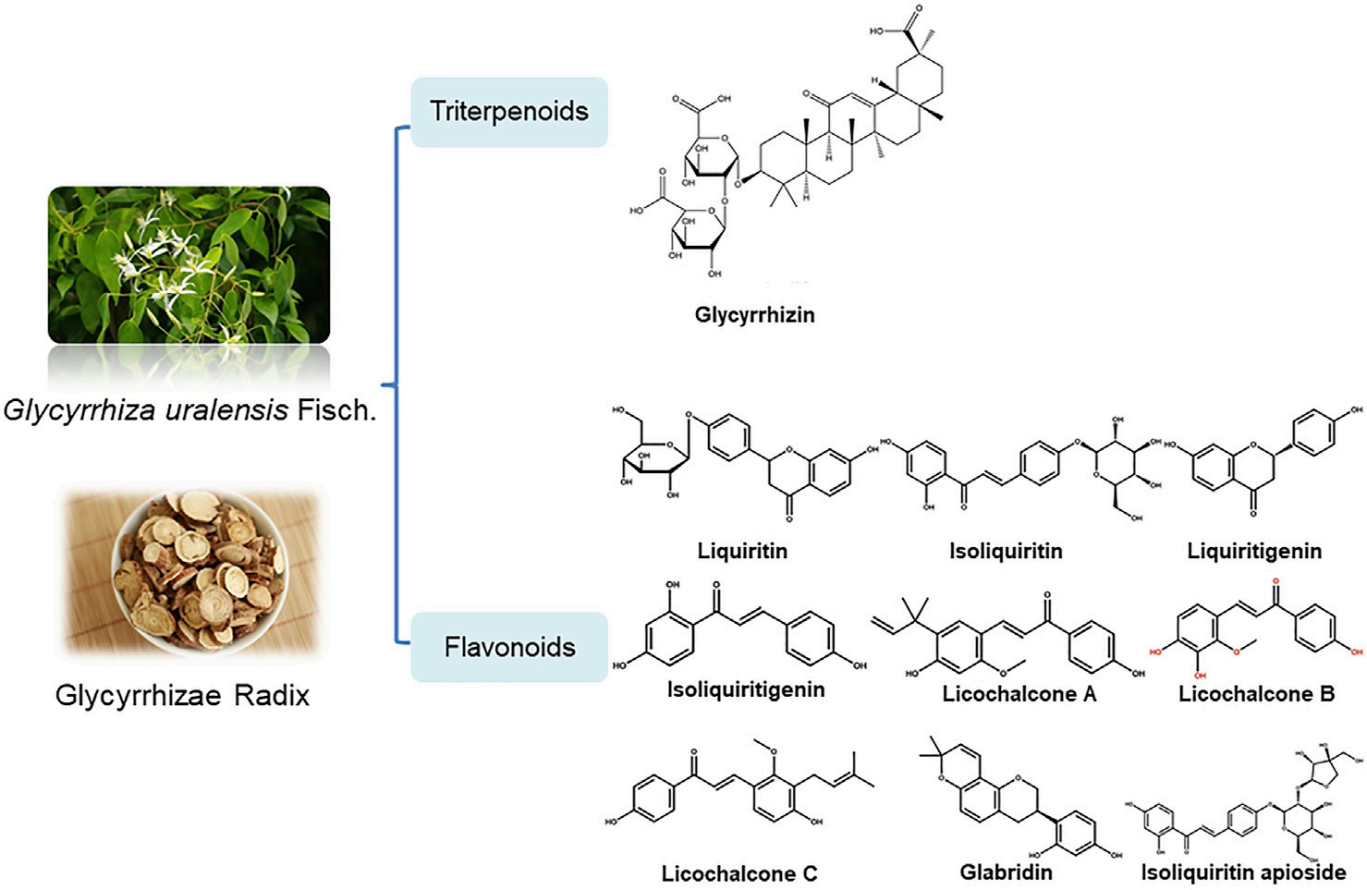
Structural formula of potential components of licorice.

Bailly et al. proposed that glycyrrhizin has a potential use in the treatment of coronavirus infection, but this article focuses on the description of the mechanism of anti-virus (mainly anti-HIV effect, anti-animal virus effect) and anti-inflammatory activity (mainly skin inflammation) of glycyrrhizin, and does not comprehensively review the related mechanism of licorice in the prevention and treatment of all potential active components of COVID-19 (Bailly and Vergoten, 2020). According to existing research findings, the pharmacological effects of licorice and natural products like glycyrrhizin, liquiritin and other active components have beneficial effects to prevent some immunological responses triggered by COVID-19. The potential mechanism could be that they play an overall regulatory role through multi-components and multi-targets to participate in biological processes (Altay et al., 2016; Yang et al., 2017; Öztürk et al., 2018; EI-Saber et al., 2020) (Table 1; Figure 1.). Therefore, the object of this review will be expanded to licorice for the prevention and treatment of all potential active components of COVID-19, to comprehensively explore the pharmacological effects and mechanism of action of licorice in potential treatment and discusses the practicability of its clinical treatments for COVID-19.

**TABLE 1:** The potential components of licorice.

Number	Component	Molecular formula	Pharmacological activity	References
1	Glycyrrhizin	C_42_H_62_O_16_	Anti-inflammatory, antiviral, antibacterial, immunomodulatory, anti-pulmonary fibrosis, and inhibition of ACE2	Altay et al. (2016); El-Saber Batiha et al. (2020)
2	Liquiritin	C_21_H_22_O_9_	Antiviral, immunomodulatory	El-Saber Batiha et al. (2020)
3	Isoliquiritin	C_21_H_22_O_9_	Anti-inflammatory, immunomodulatory	El-Saber Batiha et al. (2020)
4	Liquiritigenin	C_15_H_12_O_4_	Anti-inflammatory, antiviral, antibacterial, immunomodulatory	Öztürk et al. (2018); El-Saber Batiha et al. (2020)
5	Isoliquiritigenin	C_15_H_12_O_4_	Anti-inflammatory, antiviral, antibacterial	Yang et al. (2017)
6	Neoisoliquiritin	C_21_H_22_O_9_	Anti-inflammatory	El-Saber Batiha et al. (2020)
7	Licoflavonol	C_20_H_18_O_6_	Anti-inflammatory	El-Saber Batiha et al. (2020)
8	Isolicoflavonol	C_20_H_18_O_6_	Anti-inflammatory	El-Saber Batiha et al. (2020)
9	Licochalcone A	C_21_H_22_O_4_	Anti-inflammatory, antiviral, antibacterial, immunomodulatory	Yang et al. (2017)
10	Licochalcone B	C_16_H_14_O_5_	Anti-inflammatory, antiviral, immunomodulatory	Yang et al. (2017)
11	Licochalcone C	C_21_H_22_O_4_	Anti-inflammatory, antiviral, immunomodulatory	Yang et al. (2017)
12	Licochalcone D	C_21_H_22_O_5_	Anti-inflammatory, antiviral, immunomodulatory	Yang et al. (2017)
13	Licoricone	C_22_H_22_O_6_	Anti-inflammatory	El-Saber Batiha et al. (2020)
14	Glabridin	C_20_H_20_O_4_	Anti-inflammatory, antibacterial, vascular protection	Yang et al. (2017)
15	Glabrene	C_20_H_20_O_4_	Anti-inflammatory	El-Saber Batiha et al. (2020)
16	Glabranin	C_20_H_20_O_4_	Anti-inflammatory	El-Saber Batiha et al. (2020)
17	Uralenin	C_20_H_18_O_6_	Anti-inflammatory	El-Saber Batiha et al. (2020)
18	Licocoumarin A	C_25_H_26_O_5_	Anti-inflammatory	El-Saber Batiha et al. (2020)
19	Kanzonol R	C_22_H_26_O_5_	Antiviral	El-Saber Batiha et al. (2020)

## Pathogenesis of COVID-19

**FIGURE 2:**
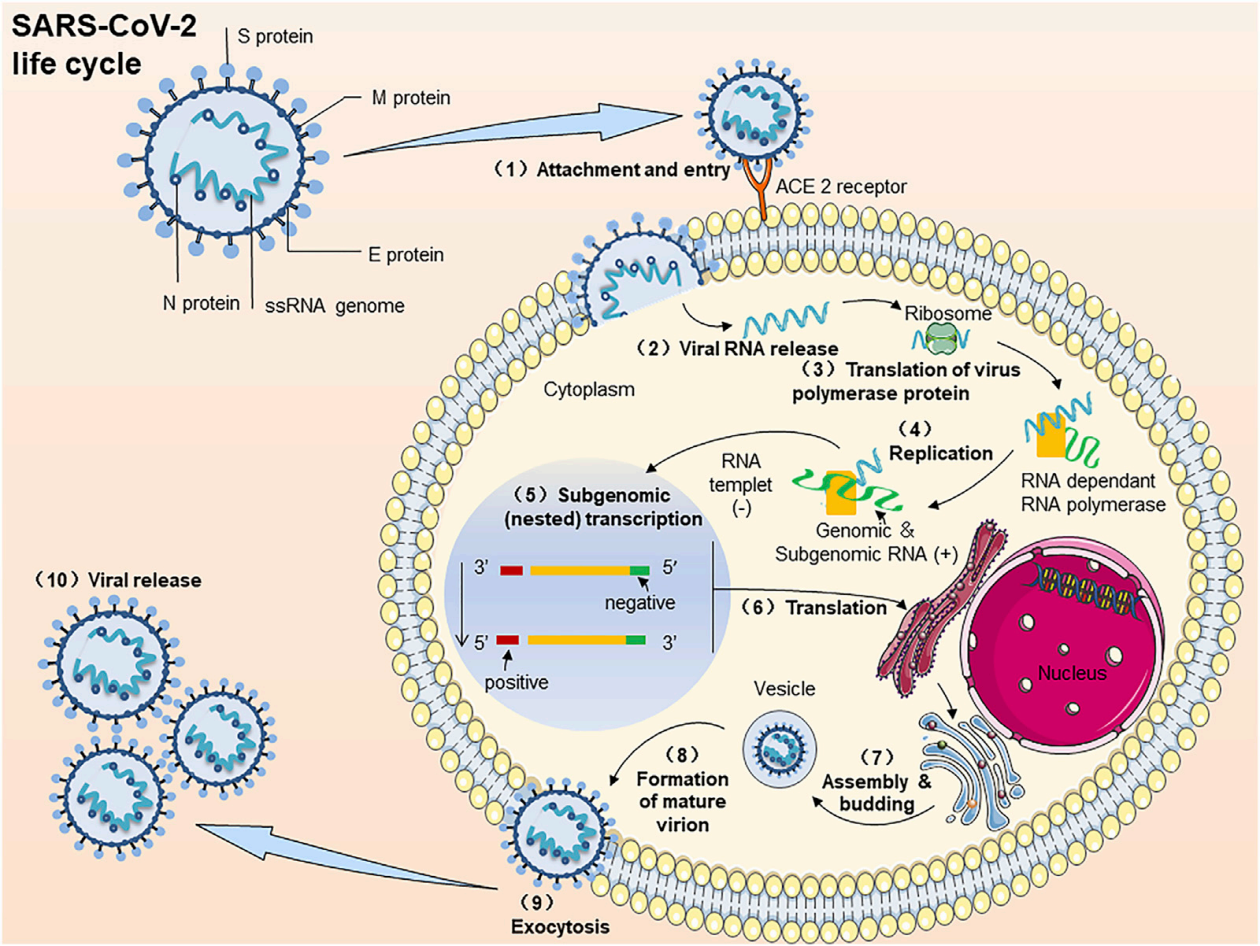
The process of SARS-CoV2 virus infection of host cells. Note: The replication cycle of SARS-CoV2 in virus-susceptible host cells: (1) ACE2 binds to the RBD of the spike protein (S) and then fuses with the host cell membrane. (2) Release the positive single-stranded RNA. (3, 4) Partially translate into SARS-CoV2 polymerase protein. (5) Transcribe. (6) The resulting subgenomic RNA translated S, M, and E proteins are transported to the ER membrane of the host cell and then bind to the nucleocapsid protein (N). (7) To-after processing in the Golgi apparatus. (8) Mature virus particles are formed and transported to the cell membrane. (10) New SARS-CoV2 particles are excreted by exocytosis (9).

COVID-19 is triggered by SARS-CoV2. Similar to SARS and MERS, SARS-CoV2 also belongs to β-coronavirus genus. New coronary pneumonia is caused by SARS-CoV2. It is a positive single-stranded, polymorphic enveloped RNA virus. The genome length is approximately 30 kb, and the particle size is approximately 100–160 nm (Shah et al., 2020). The replication cycle of SARS-CoV2 infected host cells can be divided into several key steps (Figure 2.) (Poduri et al., 2020; Ma et al., 2021): (A) Binding; (B) Membrane fusion; (C) Translation/replication; (D) Assembly and release. Specific steps include 1) The RBD of the spike protein (S) Binds to ACE2 and then fuses with the host cell membrane. 2) release the positive single-stranded RNA. (3–4) Partially translate into SARS-CoV2 polymerase protein. 5) Transcribe 6) This resulting subgenomic RNA-translated S, M, and E proteins are transported to the ER membrane of the host cell and then bind to the nucleocapsid protein (N). 7) After processing in the Golgi apparatus. 8) Mature virus particles are formed and transported to the cell membrane. (10) New SARS-CoV2 particles are excreted by exocytosis (9).

Since the emergence of SARS-CoV2, researchers have conducted in-depth studies on its genome sequence 1–3, 8, and viral protein structure 9–11. So far, studies have shown that SARS-CoV2 and SARS-CoV have many biological characteristics in common: there are 79.6% genomic sequence homology 1 and 2. In particular, both SARS-CoV2 and SARS-CoV enter the system by binding viral S protein to ACE 2 on the surface of host cells. Like SARS, MERS, and SARS-CoV2 can cause respiratory infections, leading to viral pneumonia and acute respiratory distress syndrome (ARDS). Fever, cough and fatigue are the most common clinical symptoms in patients with COVID-19. Among them, the increase of C-reactive protein (CRP), the decrease of lymphocyte count, and the rise of lactate dehydrogenase are the most common clinical abnormal values. Ground glass opacity and bilateral pneumonia are the most frequently reported findings in CT (Fu et al., 2020). A large number of experimental data showed that the plasma numbers of IL-2, IL-7, IL-10, granulocyte colony-stimulating factor, interferon-γ inducible protein, monocyte chemoattractant protein-1, macrophage inflammatory protein 1-α, and TNF-α were higher in patients with ICU (Guo et al., 2020). Nevertheless, besides respiratory symptoms, COVID-19 also produce a cytokine storm that causes the immune system to overproduce pro-inflammatory and chemokines, next causing abnormal blood coagulation in patients and thromboembolism, which results in multiple organ damage.

## Experimental Studies on Potential Treatment of COVID-19 With Licorice

### Anti-inflammatory Effects

**FIGURE 3:**
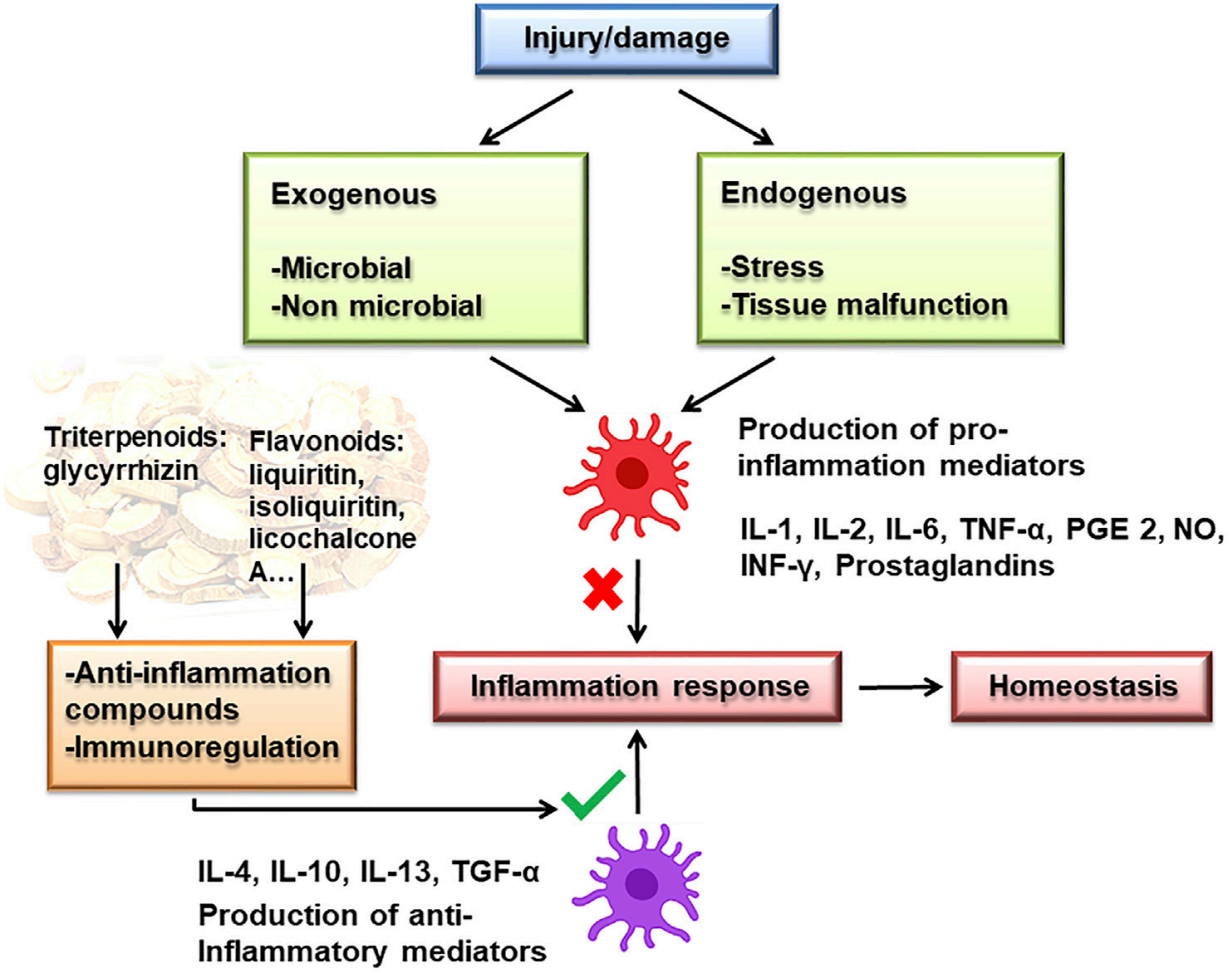
Anti-inflammatory mechanism of licorice.

The cytokine storm not only recruits more immune cells but also the homeostasis of the immune system and the function of normal cells will also be damaged, resulting in impaired ventilation function of the lungs. Inflammation is involved in many steps in COVID-19 infection, including the increase of CRP in the early stage, increase in inflammatory factors during the treatment, and sepsis in the later stage (Mehta et al., 2020). Studies have shown that licorice and its flavonoids have anti-inflammatory effects, mechanisms related to inhibiting pro-inflammatory cytokines and inflammatory mediators that participate in the MAPK signaling pathway and promote immune function (Figure 3.).

The pro-inflammatory cytokines and inflammatory mediators are significant bioactive substances that cause inflammation. One of the markers of SARS-CoV2 infection is that high numbers of IL-6 and other inflammatory cytokines lead to cytokine release syndrome (CRS), causing ARDS (Wu et al., 2020). Therefore, developing effective inflammatory mediator antagonists could inhibit the cytokine-mediated inflammatory syndrome and provide a basis for the treatment of COVID-19. Glycyrrhizin is a well-known anti-inflammatory component in licorice. It can inhibit pro-inflammatory cytokines to regulate the inflammatory response (Michaelis et al., 2011; Bhattacharjee et al., 2012; Ishida et al., 2013; Luo et al., 2013; Fu et al., 2014a; Fu et al., 2014b; Oztanir et al., 2014; Tsao and Yin, 2015; Yu et al., 2015; Li et al., 2017). During the cause of the disease, many essential signaling cascades are dysregulated, which results in hyperinflammation, hypercytokinemia, and severe diseases, such as JAK/STAT, NF-κB, TGF β, MAPK (Battagello et al., 2020; Catanzaro et al., 2020; Jose and Manuel, 2020; Matsuyama et al., 2020). For example, Wu et al. detected the phosphorylation status of Jak2 and Stat3 by specific phosphorylation antibodies. The results showed that the phosphorylation numbers of Jak2 and Stat3 increased significantly in pdx model mice, while the phosphorylation numbers of Jak2 and Stat3 decreased significantly after glycyrrhizin treatment. Glycyrrhizin can inhibit the activity of JAK/STAT signal pathway of HMGB1 upstream regulatory factor (Wu et al., 2018). Additionally, as a promising drug for treating inflammatory pain, glycyrrhizin has been shown to inhibit the high expression of lps-activated microglial high mobility group box 1 and TLR4-NF-κB pathway (Sun et al., 2018). It was found that glycyrrhizin decreased cell viability and increased apoptosis by inhibiting NF-κB signal pathway (Li et al., 2014). It has been reported that dipotassium glycyrrhizinate promotes the expression of miR146a and miR16 through the NF-κB signal pathway to inhibit proliferation and increase apoptosis in malignant glioma cells (Bonafe et al., 2019). The above studies indicate that glycyrrhizin can effectively inhibit the inflammatory response by inhibiting the synthesis and release of pro-inflammatory cytokines and inflammatory mediators, and have the possibility of becoming good drugs for the treatment of COVID-19-related diseases, with potential medicinal value.

Additionally, it is indicated that other secondary metabolites of licorice also have anti-inflammatory effects (Yang et al., 2015). Inhibition of inflammation was due to inhibition of the formation of COX-2, NO, PGE 2 and cyclooxygenase activity (Tsukahara et al., 2005; Kim et al., 2014; Jia et al., 2017). The important pathway of anti-inflammatory effect was related to the mitogen-activated protein kinase (MAPK) and NF-κB pathways (Wang D. et al., 2020). Moreover, licochalcone A, as a phenolic ketone compound, can activate the Nuclear factor-E2 related factor2 (Nrf2) and Keap1-Nrf signaling pathways to inhibit inflammation by enhancing phosphorylation of serine 349 and the expression of P 62 (Su et al., 2018). ERK and P 38 signaling pathways may play an important role in alleviating allergic airway inflammation by licorice (Chu et al., 2013). Furthermore, studies have shown that licochalcone derivatives have specific stimulation immunity (Lee JS. et al., 2015), which can suggest another mechanism of the anti-inflammatory effects of licorice is related to promoting immune function. The above studies indicate that the secondary metabolites of licorice can also effectively inhibit the inflammatory response by inhibiting the synthesis and release of pro-inflammatory cytokines and inflammatory mediators, and the regulation of relevant immune system functions is also one of the potential mechanisms of anti-inflammatory effect, with potential medicinal value.

It has been reported that licorice protects against inflammatory diseases through other anti-inflammatory mechanisms. For instance, Broncho alveolar lavage fluid (BALF) cells of COVID-19 patients were analyzed by transcriptome sequence. It was shown that chemokines such as CXCL10 and CCL-2 were released in large quantities upon SARS-CoV2 infection (Xiong et al., 2020). The administration of 3, 10, and 30 mg/kg licorice flavonoids (LF) significantly reduced the LPS-induced inflammatory cells, including neutrophils, macrophages and lymphocytes accumulation in bronchoalveolar lavage fluids (BALF), among these inflammatory cells, LF predominately inhibited neutrophil infiltration, and the maximal effect (30 mg/kg) was as comparable as dexamethasone treatment at 1 mg/kg. Consistent with its effects on neutrophil infiltration, LF treatment significantly increased LPS-induced BALF superoxide dismutase activity, and significantly decreased lung myeloperoxidase activity as well (Xie et al., 2009). Leung reviewed that the higher numbers of ACE2 in the respiratory tract of patients with chronic obstructive pulmonary disease (COPD) may increase their risk of the COVID-19 infection (Leung et al., 2020). COPD is a type of pneumonia caused by toxic particles such as cigarette smoke, fly ash, and diesel exhaust particles, which leads to severe diseases, such as chronic bronchitis and pulmonary dysfunction. Kim combined oral *Glycyrrhiza glabra* L*.* and *Acorus tatarinowii* to treat COPD. The results indicated that lung tissue pathological injury could be alleviated, and inhibit neutrophil airway inflammation more effectively by blocking the IL-17/STAT-3 pathway and regulating the expression of CXCL-2 and inflammatory cytokines (Kim et al., 2020). Additionally, 3, 10, and 30 mg/kg liquiritin apioside (LA) can inhibit TGF-β and TNF-α expression and increase anti-oxidative levels of GSH, which suggests that LA has a protective effect on pulmonary epithelial cell injury in COPD (Guan et al., 2012). Also, isoliquiritin, liquiritigenin, and isoliquiritigenin can inhibit fatty hepatitis and obesity (Kim YW. et al., 2011; Ahn et al., 2013). The mechanism involves inhibiting the inflammatory response of mouse macrophages induced by lipopolysaccharide (LPS) by inhibiting mRNA expression, iNOS and COX-2 protein, which provides a clinical reference point regarding COVID-19 patients with chronic diseases. In addition, *in vitro* studies have indicated that licorice was effective in combination with other herbs could decrease a few classical cytokines including TNF-α, IL-1β, MCP-1, and IFN. The levels of cytokines were back to normal, indicating that licorice composed with other herbs helps reduce the inflammatory level (Wang et al., 2021).

The above results show that licorice can play an anti-inflammatory role by inhibiting pro-inflammatory cytokines and inflammatory mediators, participating in MAPK-related signaling pathways and promoting immune function during COVID-19 treatment, thus inhibiting inflammation and the occurrence of high inflammatory response or cytokine storm syndrome (Wang et al., 2015). Although pharmacodynamic mechanisms and clinical studies of licorice are being discovered and applied, its curative effect needs to be further studied. Therefore, researchers can rely on the studies of COVID-19 combined with the aldosterone-like skeleton structure and anti-inflammatory solid effect of licorice to provide a more reliable basis for potential treatment in the future.

### ACE2 Inhibition

ACE2 is an indispensable protein that regulates the conversion of Ang I and Ang II into Ang 1–9 and Ang 1–7 by regulating the Ang II-Ang (1–7)-mAs axis and renin-angiotensin system, which protects the lung from ARDS and maintains normal cardiovascular and renal function. Ang II-Ang (1–7)-mAs axis, has been suggestive of potential therapy to treat inflammatory diseases, such as lung disease, cancer, diabetes, and hypertension (Fang et al., 2020). Studies have been completed to establish the ability of COVID-19 to invade and enter host cells, which mainly occurs through a spike protein in its structure along with ACE2 as the receptor (Lan et al., 2020; Zhang et al., 2020). Additionally, ACE2 is widely expressed in the respiratory tract, intestinal tract, kidney, immune cells, pancreas, arterial and venous endothelium, which may explain the origin of the main clinical symptoms of COVID-19 (Gong et al., 2012). SARS-CoV2 combined with ACE2 destroys the balance between Ang I/Ang II, and Ang (1–9)/Ang (1–7) and also increases free radicals, which leads to organ damage. Similar to SARS-CoV, SARS-CoV2 may have a high affinity for ACE2 and can infect cells through the ACE2 receptor (Xu et al., 2020). However, the expression level of ACE2 receptor differs across organ and tissue types, and ACE2 protein is highly expressed in alveolar epithelial cells and intestinal epithelial cells. This is similar to the symptoms of diarrhea and lung disease (Hamming et al., 2004). When SARS-CoV2 enters the human body, the increase in the level of Ang II stimulates angiotensin II type 1 receptor and then increases the permeability of pulmonary capillaries, leading to acute lung injury pulmonary edema and pulmonary failure. Subsequently, the virus could continue to infect the heart, liver, and kidney through the circulation of ACE2 in the blood, further triggering excessive immune responses such as Th1 and Th2 cell imbalance and producing in large numbers inflammatory cytokines. Then, the cytokine storm would eventually lead to multiple organ dysfunction syndromes (Qiao et al., 2020).

**FIGURE 4:**
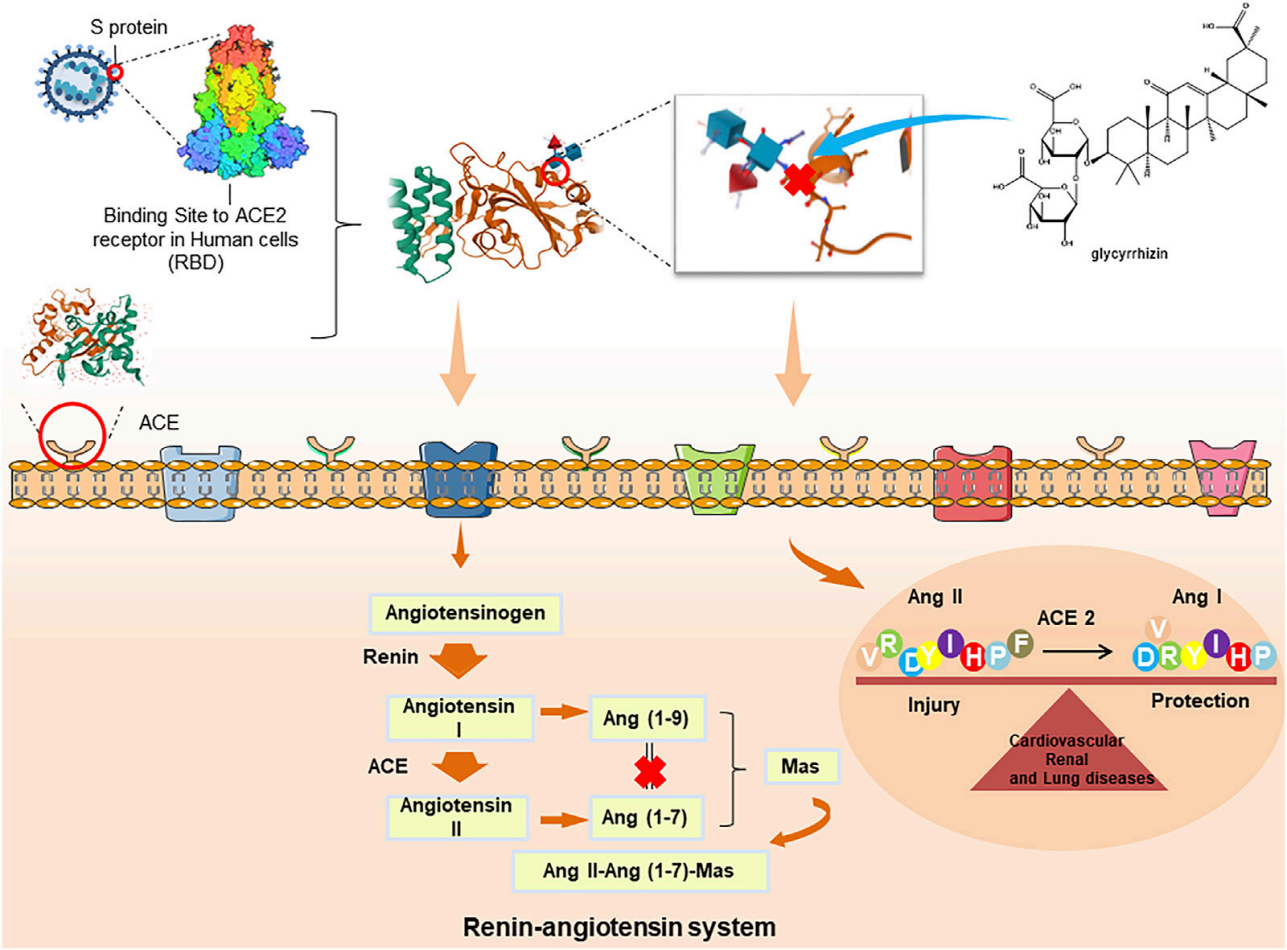
Interaction between SARS-CoV2 and ACE2 under the action of licorice.

Recent studies showed that both SARS-CoV2 and SARS coronavirus enter the cells via the ACE2 receptor, and glycyrrhizin can significantly bind to ACE2, which is worthy of further study of its potential anti-COVID-19 effects, and results showed that glycyrrhizin has no cell toxicity to mouse aorta smooth muscle cells even at high concentrations (100 μM) (Yu et al., 2020). The mechanism of glycyrrhizin against respiratory viruses may be stabilizing the SARS-COV2-S-RBD-ACE-2 complex, inhibiting the 3 CL hydrolase of COVID-19, and inhibiting the protein synthesis of the virus, which inhibits virus replication and may also be related to the improvement of inhibition of inflammation, immune regulation, up-regulation of NO expression, and protection of the host (Figure 4.). Moreover, in SARS-CoV2 infection, ACE 2 may aggravate the disease by activating NLRP3 inflammatory corpuscles in renal tubular epithelial cells catalyzed by the renin-angiotensin system (Wen et al., 2016). Shubin used the NLRP3 inflammatory body activation model to explore the regulatory effect of licochalcone A on the NLRP3 inflammatory body and its preliminary mechanism. Results showed that licochalcone A could inhibit pro-caspase-1 splicing, block caspase p20-mediated shear maturation of pro-IL-1β, and ultimately inhibit the immune-inflammatory response mediated by NLRP3 inflammatory bodies (Shubin et al., 2018). These results provide a basis for licorice in treating COVID-19 and NLRP3 inflammatory body-related diseases (Qiao et al., 2020). The above studies indicate that glycyrrhizin and the secondary metabolites of licorice can effectively inhibit the inflammatory response by inhibiting the synthesis and release of ACE 2, and have the possibility of becoming a good drug for the treatment of COVID-19-related diseases, with potential medicinal value. Licorice has the advantage of inhibiting ACE 2, but the mechanism of inhibiting ACE 2 has not been fully elucidated, and further studies are needed to promote the development of new drugs.

But it is well known that licorice is also active against many other viruses which not interact with ACE2. Recently, it is important to notice that glycyrrhizin and its metabolite 18-β-glycyrrhetinic acid as the best ligand have shown a strong binding affinity of five SARS- CoV2 proteins, SARS-CoV2 protein targets include Main protease, Papain-like protease, RNA-dependent RNA polymerase, Spike glycoprotein, Helicase, and E-Channel protein (Rehman et al., 2021). Among them, E-Channel protein shares striking functional similarities in different coronaviruses, including SARS-CoV and MERS-CoV. In addition to the essential roles of 2-E channel have been found that deletion of E channel results in attenuating SARS-CoV pathogenesis. Beyond that, E channel was also found to participate in MERS-CoV assembling, virion release, and pathogenesis. Thus, the small molecules targeting SARS-CoV2 envelope protein could also be potential broad-spectrum anti-coronavirus drugs, such as an inhibitor of E protein (Tomar et al., 2021; Wang et al., 2021).

In summary, licorice may directly or indirectly protect target organs by inhibiting the binding of the virus to ACE2, antioxidant, and anti-fibrosis effects (Ming et al., 2020). The pharmacodynamic mechanisms and clinical studies of licorice are being discovered and applied, it needs to be further studied in the treatment of COVID-19. In the future, researchers can conduct further studies of ACE inhibition on glycyrrhizin, which could lead to the development of glycyrrhizin as a potential antiviral medicine for the potential treatment of COVID-19.

### Antiviral Effects

Glycyrrhizin is inhibitor of the inflammation caused by viral and bacterial infections. Since the 1970s, it has been reported that the antiviral effect of glycyrrhizin by inhibiting the replication and infection of various DNA and RNA viruses at low concentrations without affecting the activity and proliferation of normal cells (Cai et al., 2012; Duan et al., 2015; Feng Yeh et al., 2013; Hardy et al., 2012; Matsumoto et al., 2013), or significantly inhibiting virus proliferation by activating the immune function. *In vitro* experiments showed that glycyrrhizin has strong anti-influenza effect by effectively inhibiting the replication (IC_50_ = 0.27 mg/ml) of SARS associated coronavirus strains, interfered with the cycle of adsorption, and osmotic replication of several viruses (Adianti et al., 2014; Ashfaq et al., 2011; Baltina et al., 2019; Feng Yeh et al., 2013; Huang et al., 2012; Matsumoto et al., 2013; Nomura et al., 2019; Sakai-Sugino et al., 2017; Song et al., 2014; Soufy et al., 2012; Sun et al., 2019; Wang J. et al., 2013; Zhang et al., 2012; Sun et al., 2019) against several enveloped viruses (such as EBV, HAV, HBV, HCV, HIV, HRSV, VSV, VZV) and influenza viruses including H1N1 and H5N1 (Baltina et al., 2015; Michaelis et al., 2011; Michaelis et al., 2010). The basic of these effects could be attributed to the inhibition of expression and replication of viral genes, reduction of stress, adhesion, and the binding of HMGB 1 to DNA (Moisy et al., 2012). It could also relate to serum albumin binding (Wang et al., 2020a; Wang et al., 2020b; Wang H. et al., 2020), inhibit host cell apoptosis (Wang et al., 2015) or enhance the activity of host cells (Table 2). Another widely discussed mechanism of glycyrrhizin activity is the inhibiting or changing of virus membrane fusion with host membrane (Bailly and Vergoten, 2020; Chrzanowski et al., 2021).

**TABLE 2:** Antiviral active components and their possible mechanism of virus prevention.

Component	Antiviral mechanism	Virus type	References
Glycyrrhizin	Blocking I*κ*B	CVB3	Zhang et al. (2012)
	Activating T lymphocyte proliferation	DHV	Soufy et al. (2012)
	Cutting adhesion force and stress between PMN and CCEC.	HSV	Huang et al. (2012)
	Reducing H5N1-induced production of CCL5, IL-6, and restraining H5N1-induced apoptosis	H5N1	Michaelis et al. (2010)
	Deactivating CVA16, inhibiting the virus to exertanti-EV-71 effect	CVA16, EV71	Michaelis et al. (2010); Wang et al. (2013a)
	Stimulating IFN secretion, inhibiting virus attachment, and internalization	HRSV	Matsumoto et al. (2013)

According to recent studies, the binding of glycyrrhizin and ACE2 can effectively prevent COVID-19 infection, implying that glycyrrhizin can to bind to ACE2 at its binding sites (Arg-393, Arg-559, Asp-30 and Gln-388), which suggests that glycyrrhizin has potential binding for ACE2 (Chen and Du, 2020a). For instance, Ma used the network pharmacology approach, molecular docking, and other technologies. It is important to notice that glycyrrhizin can be used as a 3 CL hydrolase inhibitor of SARS-COV2 through a series of molecular reactions in the cell membrane and cytoplasm, such as ATP binding, protein binding and enzymatic reactions to participate in the regulation and signal transduction of cell biological processes with multi-channels cooperating to show anti-COVID-19 effect (Ma et al., 2020). Nonetheless, Zhou showed that glycyrrhizin could strongly combine with SARS-COV2-S-RBD-ACE2 binding domain with SARS-COV2-S-RBD-ACE2, further, affect its stability to produce anti-SARS-COV2 (Zhou S. et al., 2020). Based on combining computer-aided drug design and biological verification, glycyrrhizin has been reported as a potential antiviral molecule. Using surface plasmon resonance technique and live cell real-time protein interaction detection technique, it was found that glycyrrhizin could directly bind to the spike protein of SARS-CoV2, thus affecting the interaction between SARS-CoV2 and ACE2 (Yu et al., 2020). Owing to low toxicity, its potential interaction with ACE2, and its antiviral effect on SARS, it is of great necessity to further study antiviral effect of glycyrrhizin. Numbers of studies indicate the promise of using various inhibitors of the fusion of viral particles with the cell plasma membrane (Tang et al., 2020), the membrane modifying activity of glycyrrhizin is described in a number of physicochemical studies and reviews. Selyutina hypothesized that the increased bioavailability of the drug by glycyrrhizin is not only due to increased solubility, but also to enhancement of drug permeability through cell membranes (Selyutina et al., 2016a). Glycyrrhizin was shown to increase the permeability (about 60%) and to decrease elasticity modulus of cell membranes (by an order of magnitude) even in micromolar concentrations (Selyutina et al., 2016b). The most intriguing feature of glycyrrhizin which might be the key factor in its therapeutic activity is the ability of glycyrrhizin to incorporate into the lipid bilayer and to increase the membrane fluidity and permeability (Selyutina and Polyakov, 2019). Furthermore, glycyrrhizin derivatives also show anti-SARS-CoV effect, which could be significantly improved by introducing 2-acetylamino-β-D-glucopyranosamine into the glycoside chain. The above studies indicate that glycyrrhizin can effectively protect host cells from viral infection by inhibiting the viral replication cycle and has the possibility of becoming a good drug for the treatment of COVID-19-related diseases, with potential medicinal value.

Also, plenty of LF has broad-spectrum antiviral effects by inhibiting the expression and replication of viral genes and inducing apoptosis of related cells. For example, licochalcone A and other chalcones, liquiritigenin, liquitin7-apigenin, isoliquiritigenin, glycyrrhizylcoumarin, glycyrrhizin opioid glycoside, quercetin, neo-isopenicillin, iso-oligosaccharide, and kanzonol Y have strong inhibitory effects on HCV (Adianti et al., 2014), HIV (Fukuchi et al., 2016), HSV-1 (Lee et al., 2017), EBV (Lee M. et al., 2015), EBOV (Fu et al., 2016), DENV (Powers and Setzer, 2016) and H1N1, influenza viruses including the new H1N1 (Dao et al., 2011). Zhu showed that glycyrrhizin can effectively inhibit SARS-CoV2, and the mechanism may be by imitating type I interferon to exert antiviral effect (ZhuJie et al., 2020). All the above studies indicate that LF can effectively exert broad-spectrum antiviral activity by inhibiting the expression of viral genes and have the possibility of becoming a good drug for the treatment of COVID-19-related diseases, with potential medicinal value.

In conclusion, these results provide evidence that a number of mechanisms could cause the beneficial effects of the active components of licorice. The basic of these effects could be attributed to the stabilization of SARS-CoV2-S-RBD-ACE2 complex and the inhibition of 3CL hydrolase of SARS-CoV2, as well as the inhibition of protein synthesis of the virus, resulting in the inhibition of viral replication. It may also be related to the improvement of immune regulation, up-regulation of NO expression, inhibition of platelet aggregation, inflammatory response and protection of host, which suggests it may be useful to develop an alternative medicine for adjuvant treating COVID-19 to some extent. However, these studies are preliminary clinical observations without rigorous trial design, and need to be verified by a large number of clinical observations with rigorous trial design in the future.

### Antibacterial and Antifungal Effects

Common in patients with COVID-19 are secondary bacterial co-infection. Thus, it is of great necessity to provide antibiotic treatment for some patients who are infected with bacteria. In addition to the primary biological activities before mentioned, licorice have antiviral effects and broad-spectrum antibacterial effects, so it may be an effective treatment for infections or secondary infections in patients with COVID-19. Studies in large numbers have indicated that licorice and glycyrrhizin, liquiritigenin, isoliquiritigenin and some flavonoids have strong antibacterial effects against Gram-positive cocci, Gram-positive bacteria, and *Bacillus cereus* (Rodino et al., 2015). The mechanism of action is related to reducing the expression of bacterial genes, the production of bacterial toxins, and inhibiting bacterial growth (Messier and Grenier, 2011; Zhou et al., 2012; Dai et al., 2013; Long et al., 2013) (Table 3).

**TABLE 3:** Antibacterial and antifungal mechanism of licorice.

Component	Antibacterial/antifungal mechanism	Type of microorganism	The range of the concentrations	References
Glycyrrhizin	Reducing the expression of key genes *SaeR* and *Hla* of MRSA virulence	*S. aureus; Enterococcus*	MIC: 62.5 μg/ml	Long et al. (2013); Schmidt et al., 2016)
	Inhibiting pathogenic bacteria and reducing the bacterial gene expression		MIC: 4–8 mg/ml	
licochalcone A and glabridin	Restraining biofilm formation and preventing yeast-hyphal transition	*C. albicans*	MIC: 6.25–12.5 μg/ml	Messier Grenier, (2011)
licochalcone E	Reducing the production of *α*-toxin	*S. aureus*	—	Zhou et al. (2012)
Liquiritigenin	Decreasing the production of *α*-hemolysin	*S. aureus*	MIC: 512 μg/ml	Dai et al. (2013)
glycyrrhizin and its derivatives	Inhibiting nutrient acquisition and affecting bacterial metabolism	*S. aureus*	Above MIC: 128 mg/L	Oyama et al. (2016)
isoliquiritigenin and liquiritigenin	Reducing the production of bacterial toxins	*S. aureus*	MIC: 50–100 μg/ml	Gaur et al. (2016)
licochalcone A	Inhibiting fungal activity especially in the glyoxylate cycle	*T. rubrum*	MIC: 11.52 μg/ml	Cantelli et al. (2017)
isobavachalcone, 4-hydroxycarotene, and kanzonol C	Inhibiting at various extents the reverse transcriptase activity	*D. barteri*	MIC <10 μg/ml	Kuete et al. (2010)
licochalcone E	Reducing the production of α-toxin	*S. aureus*	MIC: 0.3 mg/ml	Mbaveng et al. (2008)
Glabridin	Guiding fractionation against selected fungal strains	*C. albicans*	MIC: 31.25–250 mg/ml	Fatima et al. (2009)

Much of researches in antibacterial effect have found that licorice can exert antibacterial effects by inhibiting pathogenic bacteria and reducing the bacterial gene expression. For instance, 2.4 mM glycyrrhizin decreased the minimum inhibitory concentration (MIC) of gentamicin from >8 mg/L to ≤0.125 mg/L (Schmidt et al., 2016). Long investigated the antibacterial effect of glycyrrhizin on methicillin-resistant *Staphylococcus aureus* (MRSA) in a mouse model of skin infection. The results indicated that a high concentration of glycyrrhizin had a bactericidal effect on MRSA. Recently, glycyrrhizin could reduce the expression of key virulence genes of MRSA with sublethal doses, such as *Hla* and *SaeR* (Long et al., 2013). The above studies indicate that glycyrrhizin can effectively play an antibacterial role by inhibiting bacterial gene expression and has the possibility of becoming a good drug for the treatment of COVID-19-related diseases, with potential medicinal value.

Other studies have shown that the inhibition of bacterial growth is also one of the mechanisms of the antibacterial effect of licorice. As an example, it has been shown that the antibacterial effect of glycyrrhizin and its derivatives in 50 clinical patients determines the MIC of glycyrrhizin, dipotassium glycyrrhizinate, disodium succinylglycyrrhizin, disodium glycyrrhizin, stearylglycyrrhetinate, and glycyrrhetinyl stearate. The results indicated that compared with other medications, glycyrrhizin, and disodium succinyl glycyrrhizin had a stronger antibacterial effect by inhibiting amino acid metabolism and carbohydrate (Oyama et al., 2016). More importantly, licorice can also exert its antibacterial effect by inhibiting the growth of bacteria. As a typical example, liquiritigenin and isoliquiritigenin have significant inhibitory effects on Ralstoniasolanacearum and MRSA (Gaur et al., 2016). Additionally, liquiritigenin and isoliquiritigenin can help protect human lung cells from MRSA infection by reducing the production of bacterial toxins (Dai et al., 2013; Gaur et al., 2016). The above studies indicated that glycyrrhizin and the secondary metabolites of licorice can effectively inhibit bacterial fungal activation and have the possibility of becoming good drugs for the treatment of COVID-19-related diseases, with potential medicinal value.

In a retrospective analysis published in the *Lancet*, the current situation of bacterial and fungal infections in 99 patients with COVID-19 was described for the first time. Among them, 5 cases were suspected to be complicated with fungal infection and 1 case with bacterial infection (Chen et al., 2020b). Based on the experience of SARS in 2003 and the cases of invasive Aspergillus infection secondary to severe influenza, viral infection significantly increased the possibility of concurrent or secondary fungal infection and the mortality of patients (Hong et al., 2004; Vanderbeke et al., 2018). Some flavonoids have a strong antimicrobial effect against fungal and bacterial infections, which may be a potential treatment for severe pulmonary infections, potentially treating severe pulmonary infections. For instance, licochalcone A also has antimicrobial effects by inhibiting microbial growth, reducing the expression of microbial genes and the production of microbial toxins (Tsukiyama et al., 2002; Zhou et al., 2012; Hao et al., 2013). In screening new biomolecules with antifungal activity, licochalcone A showed good antifungal activity with a MIC of 11.52 μM against *Trichomonas rubrum*. Additionally, human keratinocytes’ cell activity was determined, and the IC_50_ value of licochalcone A was 30.40 μM, which suggests moderate cytotoxicity to this human cell line (Cantelli et al., 2017). Licochalcone A also exhibited activity against yeast-like fungi such as *C. albicans* at concentrations of 6.25 to 12.5 μg/ml (Messier and Grenier, 2011). Similarly, isobavachalcone, 4-hydroxycarotene, and kanzonol C have antimicrobial effects against Gram-positive and Gram-negative bacteria. The mechanism of action is to inhibit the growth of four species of fungi, six species of Gram-positive bacteria and seven species of Gram-negative bacteria (Kuete et al., 2010). Additionally, licochalcone E has an antimicrobial effect on MRSA by reducing the production of α-toxin. Consequently, it can be used to chemically synthesize new anti-Staphylococcus aureus compounds to reduce the production of toxins in MRSA (Mbaveng et al., 2008). Glabridin is the active component of *Glycyrrhiza glabra* L. root. It also inhibits yeast, *Candida albicans*, and filamentous fungi. The results showed that glabridin showed drug-resistant modification activity against *Candida albicans* mutants, and the MIC was 31.25–250 μg/mL. This is the first time to report its activity against drug-resistant mutants (Fatima et al., 2009).

From the aforementioned studies, it can be concluded that these components can reduce the expression of microbial genes, the production of microbial toxins, and inhibit microbial growth to treat related diseases. Therefore, systematic research on the antibacterial and antifungal mechanism of licorice and further developing new antimicrobial agents may provide a basis to treat COVID-19. It is suggested that the use of licorice as a complementary treatment for COVID-19 can protect patients from a bacterial infection that usually occurs after a viral infection which is the main cause for lung disease.

### Immunomodulatory Effects

The immune status of human body is closely related to the occurrence, development and prognosis of COVID-19 infection. Clinical studies have revealed that SARS-COV2 induces excessive activation of immune cells in the lungs, producing a host of inflammatory factors to form a cytokine storm, and accumulates plenty of immune cells, tissue fluid gathered in the lungs, affects the gas exchange between alveoli and capillaries, leading to hypoxemia, acute respiratory distress, and even respiratory failure. It has been recognized that effective physical immune responses play a crucial role in virus elimination and disease prevention (Chen et al., 2020b). Recently, people pour attention into the immunomodulatory effect of licorice. It has been reported that licorice not only improves immunity against virus indirectly, but also reduces the degree of inflammatory reaction and protects organ function. Licorice has the advantages of less side effects, multi-targets, multi-levels and so on. The mechanism of the regulation of cytokine release, the activity of immune cells and pulmonary vascular permeability, and influence the process and aspects of the cytokine storm (Hendricks et al., 2012; Wang Z. et al., 2013; Ma et al., 2013; Fontes et al., 2014).

Glycyrrhizin is an effective immunomodulator that regulates the immune system by acting on themitogen-activated protein kinase (MAPK) signaling pathway, Toll-like receptor signal pathway, and stimulates immune cell activity to play an immunomodulatory role, such as macrophages, NK cells. (Bordbar et al., 2012; Gong et al., 2012; Kim et al., 2012; Zhao et al., 2016; Wang B. et al., 2018; Wang BK. et al., 2018). For example, glycyrrhizin combined with the fucose-mannose ligand could inhibit the production of IL-10 in activated macrophages, enhance IL-12, and the immunomodulatory effect of glycoprotein on macrophages (Namdar Ahmadabad et al., 2020). These results suggest that glycyrrhizin can stimulate immune cells through multiple targets and pathways to regulate immune function.

Studies have shown that glycyrrhizin can enhance the immune status by regulating the proliferation of Treg cells (Hendricks et al., 2012; Mitra Mazumder et al., 2012; Han et al., 2017; Wang et al., 2017). Among them, Treg cells inhibit T cells, antigen-presenting cells, and reduce the production of pro-inflammatory cytokines and antibody secretion, which can reflect up-regulation of immune function. Therefore, it is of great necessity that Treg cells prevent autoimmunity and control the immune response. Consequently, selectively increasing Treg cells *in vivo* have extensive therapeutic significance for autoimmune and inflammatory diseases. Other studies have shown that isoliquiritigenin and naringenin are also effective components that regulate the immune response induced and suppressed by Treg cells, and can significantly promote the proliferation of Treg cells in mice and that these two flavonoids can induce more Treg cells at lower doses than glycyrrhizin (Guo et al., 2015). These results suggest that glycyrrhizin can enhance immune function by regulating immune cells.

Licorice polysaccharide is a good immune enhancer, which can significantly improve the specific and non-specific immunity of the body, and activate the immune system by promoting the mature differentiation and reproduction of immune cells (such as lymphocytes and macrophages). For example, Ayeka reviewed that licorice polysaccharide can regulate the immunity of tumor-bearing BALB/c mice, significantly inhibit tumor growth, and increase the index of immune organs, such as CD4^+^ and CD8^+^ T cells compared to a saline group with a significant increase and decrease respectively (Ayeka et al., 2017). Min found that licorice polysaccharide was given to mice for 14 days. The delayed type hypersensitivity induced by dinitrofluorobenzene in mice was determined. The contents of IL-2 and TNF-α in blood and the effect of licorice polysaccharide on cellular immune function of mice were detected by radioimmunoassay. The results showed that licorice polysaccharide could enhance the delayed type hypersensitiity reaction and increase the contents of IL-2 and TNF-α in blood (Min et al., 2009). These results suggest that licorice polysaccharide can enhance the immune function of mice by promoting the maturation and differentiation of immune cells.

The above results demonstrate the immunomodulatory effects of licorice, which suggests that it can be used as a candidate for the development of new immunomodulatory medicine. However, the study of immunomodulatory effects of licorice is limited. Therefore, further reliable data are needed to confirm the immunomodulatory effects of licorice.

### Anti-Pulmonary Fibrosis Effects

Following effective, comprehensive treatment, the pulmonary lesions of COVID-19 patients usually recover on their own. However, some COVID-19 patients develop pulmonary fibrosis after rehabilitation (Matsuyama et al., 2020). Hence, it is worth pouring attention to the occurrence of PF in patients with COVID-19, given that early interventions may help avoid this condition. PF is a severe lung disease characterized by excessive accumulation of extracellular matrix (ECM) (Rajasekaran et al., 2015; Bardou et al., 2016). Inflammatory cell infiltration, hyperemia, and edema are predominant in the early stage, followed by alveolar epithelial cells, AEC injury, and ECM-producing cells, including excessive production of ECM, abnormal proliferation of fibroblasts (Fb), which results in progressive scarring and loss of pulmonary function.

Licorice also has a protective effect on lung tissue (Liu et al., 2019). On the one hand, glycyrrhizin can reduce local fibrosis and pulmonary edema induced by bleomycin, slightly thicken the pulmonary interstitium, and significantly reduce the content of Col-I and Hyp in lung tissue (Gao et al., 2015). However, glycyrrhizin can down-regulates AEC markers (E-cadherin) in lung tissue, significantly inhibited the increase of malondialdehyde, myeloperoxidase, transforming growth factor-Smad1, p-Smad3, p-β2 and interstitial cell markers induced by bleomycin (Gao et al., 2015). Li showed that glycyrrhizin can reduce bleomycin-induced inflammation and collagen deposition in the lungs of mice. The anti-PF effects of glycyrrhizin are considered related to phenotypic regulation and deviation of mononuclear macrophages and down-regulation of TGF-β1 expression in lung tissue (Li et al., 2017). In clinical studies, intravenous infusion of glycyrrhizin Diamine (300 mg/day for 4 weeks) combined with its capsule (150 mg/day for 5 months) significantly reduced the numbers of type III procollagen and serum hyaluronic acid in patients with PF, with mild side effects, such as elevated blood pressure, edema, palpitation (Hsieh et al., 2012).

The above results suggest that licorice can improve bleomycin-induced PF, inflammatory reaction, oxidative stress, down-regulate the TGF-β signaling pathway and epithelial-mesenchymal transition (EMT), inhibit Fb migration and proliferation, promote Fb apoptosis, especially in ECM-induced PF, and that its mechanism is closely related to its anti-inflammatory effect.

### Protection of Other Organs

A recent study published in *the Lancet* found that blood vessel is a direct target of SARS-CoV2 (Varga et al., 2020). If a blood clot, formed by the damage to the inner wall of the blood vessel, it will become loose as the blood circulates to the brain and lungs, eventually leading to a stroke or pulmonary embolism in patients with COVID-19. Licorice can protect vascular endothelial cells by inhibiting the adhesion, migration, and proliferation of related endothelial cells (Kim JM. et al., 2011; Tan et al., 2014).

As mentioned above, there is an increased prevalence and mortality due to pneumonia in COVID-19 patients with chronic diseases such as diabetes (McDonald et al., 2014; Pearson-Stuttard et al., 2016; Li et al., 2019). Studies have confirmed that liquiritigenin, isoliquiritigenin, licochalcone A, isobavachalone, glabridin, and kanzonol C have protective effects on the heart, liver, and kidney (Park et al., 2012; Wu et al., 2013; Gaur et al., 2014; Yao et al., 2014). The mechanism of the inhibition of the MAPK signaling pathway, NF-κB signaling pathway, has a protective effect on myocardial fibrosis. Additionally, flavonoids are inhibitors used to treat obesity, such as licochalcone A, isobavachalone and kanzonol C (Zhang et al., 2016; Xie, 2017; Lee et al., 2018).

The above results show that patients with COVID-19, who may have different degrees of multiple organ failure, such as lung, heart, liver, and kidney diseases, could benefit from the wide range of pharmacological activities of licorice. So, based on the existing research, future studies that examine the efficacy, dose-effect relationship, action mechanism, and target, adverse reactions, and so on, could provide a safer and more reliable basis for the clinical application of treatments for COVID-19.

## Clinical Studies on the Potential Treatment of COVID-19 With Licorice

Licorice can play an auxiliary role in the clinical treatment of COVID-19 and comprehensively relieve the adverse symptoms of patients (Table 4). In a clinical study, Zhou (Zhou, et al., 2020c) conducted a clinical observation on the treatment of COVID-19 patients with Glycyrrhizin Diamine. The patients in the control group were treated according to the “diagnosis and treatment of pneumonia caused by novel coronavirus (trial version 5),” and the patients in the observation group were treated with glycyrrhizin Diamine enteric-coated capsule (150 mg, 3 times/d, for 2 weeks). The results showed that the serum numbers of CRP, IL-4, and TNF-α in the observation group were significantly lower than those in the control group, while the numbers of CD3^+^, CD4^+^, CD8^+^, CD4^+^/CD8^+^ in the observation group were significantly higher than those in the control group, and the prevalence of adverse reactions [ 15.38% (8/52) vs. 28.85% (15/52) ] of the observation group were significantly lower than that of the control group (*p* < 0.05). Glycyrrhizin Diamine has significant clinical efficacy and high safety in the treatment of common COVID-19 patients, which can inhibit the inflammatory reaction and improve immune function. Xi (Xi et al., 2020) used Abidol combined with Glycyrrhizin Diamine Enteric-coated Capsules treated COVID-19 patents. WBC count, N%, erythrocyte sedimentation rate, PCT and other infectious indicators, CRP, IL-6 and other inflammatory indicators as well as liver and kidney function, adverse reactions and other data were collected before and after treatment. The differences of various indicators before and after treatment were analyzed to elucidate the efficacy and safety of Abidol combined with Glycyrrhizin Diamine Enteric-coated Capsules in the treatment of COVID-19. Results showed the main clinical symptoms of 46 patients were low-grade fever, cough and fatigue. Compared with that before treatment, the symptoms of patients were significantly improved after treatment (*p* < 0.05). WBC count, N%, erythrocyte sedimentation rate and other infectious indicators were decreased, of which N% had statistical significance (*p* < 0.05). Lymphocyte count was significantly increased (*p* < 0.05). CRP, IL-6, PCT and other inflammatory indicators were decreased, of which IL-6 had statistical significance (*p* < 0.05). Albumin content, ALT were significantly increased (*p* < 0.05), other liver and kidney function indicators were not significantly changed. No nausea, vomiting and other related drug adverse reactions were reported, AST, ALT increased in 3 cases, ALT alone increased in 5 cases. The overall cure rate was 63.04%, and the overall response rate was 78.26%. It suggested that Abidol combined with Glycyrrhizin Diamine Enteric-coated Capsule in the treatment of COVID-19 has reliable clinical efficacy, which can effectively reduce the inflammatory response in patients with fewer adverse reactions, high safety, is a feasible choice for the treatment of new coronary pneumonia. In another clinical study, Ding treated COVID-19 with Glycyrrhizin Diamine combined with troxerutin and vitamin C. The results showed that the body temperature of the patients began to drop and gradually returned to normal on the second and third day of treatment, and then all improved and recovered within 5 days (http://www.chictr.org.cn/). Additionally, some clinical studies on SARS treatment have shown that licorice has potential efficacy in the development of COVID-19 possible treatment (Cinatl et al., 2003). In 2004, it was reported that glycyrrhizin could be used in the treatment of SARS (Ling, et al., 2004; Yuping, et al., 2004). For example, Chi divided 60 cases of SARS into two groups on average: the treatment group was treated with compound glycyrrhizin, the control group was treated with conventional treatment. The appearing time, site, scope, and dynamic changes in the pulmonary lesions on chest radiograms were compared between two groups. Results showed that the average period from peak to 50% improvement of lesion in X-ray manifestations was shorter in group I than in group II. In the restoration stage, more patients had their X-ray findings absorbed in group I compared with the patients in group II. Compound glycyrrhizin had little influence on the White Blood Cells, blood sugar, and electrolytes. It showed that glycyrrhizin may be a promising medicine against SARS with fewer side effects (Chihong, et al., 2004).

**TABLE 4:** Clinical studies on the potential treatment of COVID-19 with licorice.

Medicine	Subject	Study design	Length	Treatment result	References
Abidol combined with Glycyrrhizin Diamine Enteric-coated Capsules	46 patients with COVID-19 (15 males and 31 females)	Randomized, Control group	—	Compared with that before treatment, the symptoms of patients were significantly improved after treatment (*p* < 0.05). WBC count, N%, erythrocyte sedimentation rate and other infectious indicators were decreased, of which N% had statistical significance (*p* < 0.05). Lymphocyte count was significantly increased (*p* < 0.05). CRP, IL-6, PCT and other inflammatory indicators were decreased, of which IL-6 had statistical significance (*p* < 0.05). Albumin content, ALT were significantly increased (*p* < 0.05), other liver and kidney function indicators were not significantly changed. No nausea, vomiting and other related drug adverse reactions were reported, AST, ALT increased in 3 cases, ALT alone increased in 5 cases. The overall cure rate was 63.04%, and the overall response rate was 78.26%	Xi et al. (2020)
Diammonium Glycyrrhizinate	104 novel coronavirus pneumonia patients (60 males and 45 females)	Randomized, Control and observation group	2 weeks	The cure rate, markedly effective rate and total effective rate of the observation group were 19.23, 28.85 and 61.54% respectively, which were significantly higher than those of the control group (7.69, 17.31 and 40.38%) (*p* < 0.05). After treatment, the average levels of serum CRP, IL-4 and TNF-α water k in the observation group were significantly lower than those in the control group, while the levels of CD3^+^, CD4^+^, CD8^+^ and CD4^+^/CD8^+^ in the observation group were significantly higher than those in the control group. The incidence of adverse reactions in the observation group (15.38%) was significantly lower than that in the control group (28.85%) (*p* < 0.05)	Zhou et al. (2020b)
Diammonium Glycyrrhizinate	Vero cells	—	72–96 h	Expression of viral antigens was much lower in cultures treated with 1,000 mg/L of glycyrhizin than in any other culture; high concentrations of glycyrthizin (4,000 mg/L) completely blocked replication of the virus	Cinatl et al. (2003)
Lianhuaqingwen capsule	284 patients (142 each in treatment and control group)	Randomized, Control and observation group	14 days	The recovery rate was significantly higher in treatment group as compared with control group (91.5 vs. 82.4%, *p* = 0.022). The median time to symptom recovery was markedly shorter in treatment group (median: 7 vs. 10 days, *p* < 0.001). Time to recovery of fever (2 vs. 3 days), fatigue (3 vs. 6 days) and coughing (7 vs. 10 days) was also significantly shorter in treatment group (all *p* < 0.001). The rate of improvement in chest computed tomographic manifestations (83.8 vs. 64.1%, *p* < 0.001) and clinical cure (78.9 vs. 66.2%, *p* = 0.017) was also higher in treatment group. However, both groups did not differ in the rate of conversion to severe cases or viral assay findings (both *p* > 0.05)	Hu et al. (2020)
Lianhuaqingwen capsule	42 patients (21 subjects in the treatment group and 21 subjects in the control group)	Randomized, Control and observation group	—	Compared with the control group, patients in the treatment group had the higher clinical effect, including the disappearance rate of fever (85.7 vs. 57.1%, χ^2^ = 4.200, *p* = 0.040), the disappearance rate of cough (46.7 vs. 5.6%, *p* = 0.012), the disappearance rate expectoration (64.3 vs. 9.1%, *p* = 0.012), the disappearance rate of shortness of breath (77.8% vs. 0, *p* = 0.021), and the duration of fever [(4.6 + 3.2)d vs. (6.1 + 3.1)d, *p* = 0.218]	

At present, there are few clinical studies on licorice in the potential treatment of COVID-19, and there are many studies on the compound prescription with licorice as the main component. For example, LianhuaQingwen capsule, which is composed of *Forsythia suspensa*, *Flos lonicerae*, licorice and other 13 herbs, can inhibit virus replication, cause changes in virion shape and inhibit the expression of inflammatory factors in host cells to play the role of anti-novel coronavirus effect. The latest research shows that LianhuaQingwen capsule can inhibit the effect of COVID-19 *in vitro* and significantly relieve the symptoms of fever, cough, and fatigue in patients with COVID-19 (Hongrong et al., 2020). Hu observed the clinical efficacy of LianhuaQingwen capsule in the treatment of COVID-19. The treatment group was treated with usual treatment combined with LianhuaQingwen capsule (4 tablets, 3 times/d), while the control group was treated with usual treatment. The results showed that after 14 days of treatment in the LianhuaQingwen treatment group, the recovery rate of the LianhuaQingwen capsule treatment group was 91.5%, which was significantly higher than that of the control group (82.4%). And the median cure time for fever, fatigue, cough, and other symptoms in LianhuaQingwen capsule treatment group was significantly shortened (Hu et al., 2020). LianhuaQingwen capsule can be considered to improve the clinical symptoms (fever, dry cough and fatigue) of COVID-19, which provide a basis for the application of LianhuaQingwen combined with existing techniques in the potential treatment of COVID-19. Yao observed the clinical effect of LianhuaQingwen capsule in the treatment of COVID-19. The treatment group was treated with routine treatment combined with LianhuaQingwen granule, and the control group was treated with routine treatment. The results showed that When compared with the control group, patients in the treatment group had higher clinical effect, including the disappearance rate of fever (85.7 vs. 57.1%, χ^2^ = 4.200, *p* = 0.040), the disappearance rate of shortness of breath (77.8% vs. 0, *p* = 0.021), and the duration of fever [(4.6 ± 3.2)d vs. (6.1 ± 3.1)d, *p* = 0.218 ]. It is suggested that the drug has a certain clinical application value in improving the symptoms of COVID-19 patients, relieving the disease, and shortening the course of disease (Kaitao et al., 2020).

## Conclusion

To sum up, licorice plays a synergistic role in regulating the symptoms of COVID-19 patients through multi-components and multi-targets, and participates in biological processes such as immunity, anti-inflammation and signal transduction, suppresses the production of cellular endotoxin, balances immunity, eliminates inflammation, avoids or alleviates inflammatory storms, and has a good clinical effect on the prevention and treatment of COVID-19. Among them, glycyrrhizin and glycyrrhetinic acid interact with ACE 2, spike protein, host transmembrane serine protease 2 and 3-chymotrypsin-like cysteine protease to alleviate the common symptoms of COVID-19 patients, such as pulmonary inflammation and liver and kidney injury. Liquiritin can relieve fever, cough, fatigue and other symptoms of COVID-19 patients to some extent by stimulating type I interferon. Oral licorice preparation (150mg/, 3 times / d for 2 weeks) could improve the symptoms of low fever, cough and fatigue in patients with mild to moderate COVID-19 (the overall effective rate ≥ 60%) (Table 5). It is suggested that different components can play a synergistic effect on the prevention and treatment of COVID-19. The first edition of Chinese Pharmacopoeia 2020 stipulates that licorice contains no less than 2.0% glycyrrhizin and 0.1% ∼ 0.2% glycyrrhetinic acid. The common clinical dose of glycyrrhizin is less than 100 mg (equivalent to 5 g licorice at the effective dose), and the common dose of glycyrrhetinic acid is 250 ∼ 500 mg (equivalent to 125 ∼ 250 g licorice at the effective dose), suggesting that licorice meets the formability of the drug preparation. At the same time, it meets the requirements of conventional drug dosage and meets the needs of clinical drug use.

At present, the clinical trial of licorice on the prevention and treatment of COVID-19 is mainly based on the improvement of inflammatory factors, suggesting that licorice and its related preparations can significantly improve the increase of inflammatory factors. However, this index is not enough to make an accurate diagnosis of the clinical symptoms of patients with COVID-19, the clinical index is too single, lack of respiratory diseases, virus metastasis rate and other indicators. Therefore, for the observation of etiology and related blood test indicators (such as serological detection), the diagnostic efficiency of multi-index combined detection is very important.

At present, most of them are based on experimental basic research, so there is a lack of sufficient clinical research. It is believed that with the deepening of clinical research, its more reasonable dosage, more accurate evaluation index and mode of administration will be deeply analyzed. At the same time, it will provide the basis for the new use and clinical application of licorice.

## Glossary

ACE2: angiotensin-converting enzyme 2
AEC: alveolar epithelial cell
ARDS: acute respiratory distress syndrome
BALF: broncho alveolar lavage fluid
COPD: chronic obstructive pulmonary diseases
CRP: C-reactive protein
CRS: cytokine release syndrome
CVA 16: coxsackie virus A16
CYP450: cytochrome P450
DENV: dengue virus
DHV: duck hepatitis virus
EBOV: ebola virus
EBV: epstein-barr virus
ECM: extracellular matrix
EMT: epithelial-mesenchymal transition
EV 71: enterovirus 71
Fb: Fibroblast
LA: liquiritinapioside
LF: licorice flavonoids
LPS: lipopolysaccharides
MAPK: mitogen-activated protein kinase
MARV: marburg virus
MERS: middle east respiratory syndrome
MIC: minimum inhibitory concentration
MRSA: methicillin-resistant *Staphylococcus aureus*
Nrf2: nuclear factor-E2 related factor2
HAV: hepatitis A virus
HBV: hepatitis B virus
HCV: hepatitis C virus
HIV: human immunodeficiency virus
HRSV: human respiratory syncytial virus
HSV-1: herpes simplex virus
PF: pulmonary fibrosis
RBD: receptor binding domain
ROS: reactive oxygen species
SARS: severe acute respiratory syndrome
VSV: vesicular stomatitis virus
VZV: varicella zoster virus
